# Reassessment of Blood Gene Expression Markers for the Prognosis of Relapsing-Remitting Multiple Sclerosis

**DOI:** 10.1371/journal.pone.0029648

**Published:** 2011-12-27

**Authors:** Michael Hecker, Brigitte Katrin Paap, Robert Hermann Goertsches, Ole Kandulski, Christian Fatum, Dirk Koczan, Hans-Peter Hartung, Hans-Juergen Thiesen, Uwe Klaus Zettl

**Affiliations:** 1 Steinbeis Transfer Center for Proteome Analysis, Rostock, Germany; 2 Department of Neurology, University of Rostock, Rostock, Germany; 3 Institute of Immunology, University of Rostock, Rostock, Germany; 4 Department of Neurology, Heinrich Heine University, Düsseldorf, Germany; University of Muenster, Germany

## Abstract

Despite considerable advances in the treatment of multiple sclerosis, current drugs are only partially effective. Most patients show reduced disease activity with therapy, but still experience relapses, increasing disability, and new brain lesions. Since there are no reliable clinical or biological markers of disease progression, long-term prognosis is difficult to predict for individual patients. We identified 18 studies that suggested genes expressed in blood as predictive biomarkers. We validated the prognostic value of those genes with three different microarray data sets comprising 148 patients in total. Using these data, we tested whether the genes were significantly differentially expressed between patients with good and poor courses of the disease. Poor progression was defined by relapses and/or increase of disability during a two-year follow-up, independent of the administered therapy. Of 110 genes that have been proposed as predictive biomarkers, most could not be confirmed in our analysis. However, the G protein-coupled membrane receptor GPR3 was expressed at significantly lower levels in patients with poor disease progression in all data sets. GPR3 has therefore a high potential to be a biomarker for predicting future disease activity. In addition, we examined the IL17 cytokines and receptors in more detail and propose IL17RC as a new, promising, transcript-based biomarker candidate. Further studies are needed to better understand the roles of these receptors in multiple sclerosis and its treatment and to clarify the utility of GPR3 and IL17RC expression levels in the blood as markers of long-term prognosis.

## Introduction

Multiple sclerosis (MS) is a chronic, progressive and disabling immune-mediated disorder of the central nervous system. Genetic susceptibility, environmental exposure and immune dysregulation play key roles in the pathogenesis of this disease [1]–[3]. Clinical heterogeneity, alternating periods of worsening/recovery and incomplete understanding of the autoreactive inflammatory processes make diagnosis and treatment of MS challenging. Currently, different immunomodulatory medications allow for control of the severity and progression of the disease. Beta-interferons (IFNs), along with glatiramer acetate (GA), are first-line drugs in the treatment of relapsing-remitting MS (RRMS), which is the most common MS phenotype [4], [5]. The course of MS in a particular patient depends on both individual disease activity and response to treatment, and is typically monitored by evaluating the rate and severity of relapses, the increase of disability (using, e.g., the expanded disability status scale, EDSS), and changes in magnetic resonance imaging (MRI). However, our ability to predict long-term progression and therapy outcome is limited. In clinical and pharmacogenomic studies, treated patients are often categorized into “responders” and “non-responders”, mostly depending on their relapse rate, EDSS and MRI. However, the term “non-responder” is somewhat misleading and does in practice not generally implicate the discontinuation of the treatment. Others, therefore, distinguish “stable” and “breakthrough disease”, the latter meaning that disease activity is present despite therapy [6]. Here, we prefer to discern patients as having a “good” or “poor” disease course. Accordingly, a patient with a “poor” course is characterized by a severe disease progression, which may be, but is not necessarily, causally related to a less effective response to a drug.

As of today, the individual course of MS is more or less unpredictable, and clinical or laboratory markers that prognosticate either favorable or detrimental therapy outcomes still need to be established. In general, females and younger patients tend to have a slower disability progression [7]. Clinical parameters that correlate with poor prognosis include more severe initial symptoms, incomplete recovery from the first attack, and a short interval between the first and second attacks [8], [9]. MRI measures, such as T2 lesion volume, are useful early in the disease [10], but later on, they have only a weak predictive strength [11], [12]. In recent years, many efforts have therefore been devoted to identifying molecular biomarkers in blood or cerebrospinal fluid (CSF) to better monitor disease evolution, estimate individual disease activity, and predict clinical improvement/worsening and therapy response [13]. The most debated biomarkers are drug-neutralizing antibodies (NAbs), which are often associated with the loss of the clinical efficacy of the medication [4]. Up to one-third of IFN-treated patients develops antibodies against the drug, depending on the IFN-beta product used [14]. IFN NAb positivity can be evaluated indirectly by measuring the capacity of IFN to induce the gene expression of MX1 [15]. Low MX1 induction, i.e. presence of NAbs, predicts the risk of both new relapses and increase of disability [16]-[18]. However, routine IFN NAb testing is still rarely performed in clinical practice.

Several transcriptional and protein profiling studies have been carried out in the field of MS, particularly to elucidate short- and long-term gene regulatory effects of IFN-beta treatment [19], [20]. Four gene expression microarray and seven RT-PCR studies, as well as seven protein analyses, described blood-based biomarkers that may allow for the long-term prognosis of MS (Tables 1 and 2) [21]–[38]. In these 18 studies, mRNA or protein levels were evaluated at a single time point in a group of mostly RRMS patients. Some authors discovered further potentially predictive markers in the blood by comparing changes in gene expression during IFN-beta treatment between clinical responders and non-responders [26], [35], [36], [39]–[43]. Others again proposed biomarkers of short-term clinical changes [44]–[46], e.g., high MMP9 and low TIMP1 serum protein levels were observed 1 month prior to the appearance of new gadolinium-enhancing lesions [46]. Several findings suggest that individual differences in endogenous type I IFN activity correlate with different long-term therapeutic outcomes to IFN-beta administration [25], [26], [37], [47], [48]. An interesting study was published by Axtell et al., who observed elevated pre-treatment serum concentrations of both IFN-beta and IL17F in a subgroup of IFN-beta non-responders [22], and later incorporated IL7 into this setting [33]. Further molecular markers indicative of MS progression have been proposed, e.g., high protein levels of CXCL13 and TNF in CSF were associated with an unfavorable prognosis [49], [50], high vitamin D levels were shown to reduce the hazard of relapse [51], single-nucleotide polymorphisms at the intronic regions of GPC5 [52] and alleles at the HLA-DRB1 locus have been linked to poor disease course [53], anti-alpha-glucose-based glycan IgM antibodies in serum were shown to confer a higher risk for relapses [54], and intrathecal IgM synthesis has been related to the onset of new relapses and worse EDSS changes [55], [56].

**Table 1:** Studies proposing blood biomarkers for MS prognosis.

Publication	Study Characteristics	Clinical Groups	Number of Genes	List of Genes
Achiron et al., Clin Exp Immunol, 2007 [21]	**Patients:** 53 RRMS, **Treatment:** IFN-â-1a im or sc, **Sample:** PBMC RNA, **Platform:** Affymetrix HG-U133 A and -U95	**Good:** no progress in neurological disability and no relapse in 2-year follow-up, **Poor:** progress with and without relapses	**Suggested:** 29, **Affymetrix custom:** 25, **Affymetrix original:** 56	ADD1, C19orf29, CA11, CCL17, CD44, CRYGD, DNM1, DR1, GNMT, GPR3, GSTA1, HAB1, HSPA8, IGLJ3, IL3RA, KLF4, KLK1, MUC4, NINL, ODZ2, PTN, RRN3, S100B, TOP3B, VEGFB, (COL11A2, IGLV2-23, TPSB2, TRB@)
Axtell et al., Nat Med, 2010 [22]	**Patients:** 26 RRMS, **Treatment:** IFN-â-1a im or sc, IFN-â-1b sc, **Sample:** serum proteins, **Platform:** Multiplex Bead Analysis	**Good and Poor:** not exactly declared; based on the number of relapses and steroid interventions within 2-year follow-up	**Suggested:** 2, **Affymetrix custom:** 2, **Affymetrix original:** 1	IL17F, **IFNB1**
Baranzini et al., PLoS Biol, 2005 [23]	**Patients:** 52 RRMS, **Treatment:** IFN-â-1a im or sc, IFN-â-1b sc, **Sample:** PBMC RNA, **Platform:** real-time RT-PCR	**Good:** no relapse and no EDSS increase after 2-year follow-up, **Poor:** two or more relapses and EDSS increase of at least one point	**Suggested:** 12, **Affymetrix custom:** 11, **Affymetrix original:** 29	CASP2, CASP3, CASP7, CASP10, CFLAR, IL12RB1, IL4R, IRF2, IRF4, MAP3K1, STAT4, (IRF6)
Bartosik-Psujek and Stelmasiak, Clin Neurol Neurosurg, 2006 [24]	**Patients:** 29 RRMS, **Treatment:** IFN-â-1a im, IFN-â-1b sc, **Sample:** serum proteins, **Platform:** ELISA	**Good:** fewer relapses during the 2 years on therapy than during the 2 years before therapy or an improved EDSS, **Poor:** Unchanged or higher relapse rate and EDSS	**Suggested:** 1, **Affymetrix custom:** 1, **Affymetrix original:** 1	IL10
Bustamante et al., Ann Neurol, 2011 [25]	**Patients:** 85 RRMS, **Treatment:** IFN-â-1a im or sc, IFN-â-1b sc, **Sample:** monocyte surface proteins, PBMC RNA, **Platform:** real-time RT-PCR, FACS	**Good:** no relapse and no EDSS increase after 2-year follow-up, **Poor:** one or more relapses and EDSS increase of at least one point	**Suggested:** 4, **Affymetrix custom:** 4, **Affymetrix original:** 6	**IFNAR1**, **IFNB1**, **IL1B**, IRAK3
Comabella et al., Brain, 2009 [26]	**Patients:** 47 RRMS, **Treatment:** IFN-â-1a im or sc, IFN-â-1b sc, **Sample:** PBMC RNA, monocyte surface proteins, **Platform:** Affymetrix HG-U133 Plus 2.0, FACS	**Good:** no relapse and no EDSS increase after 2-year follow-up, **Poor:** one or more relapses and EDSS increase of at least one point	**Suggested:** 28, **Affymetrix custom:** 26, **Affymetrix original:** 34	ATF3, CCR1, CXCL10, CXCL2, EGR3, HERC5, IER3, IFI44, IFIT1, IFIT2, IFIT3, **IFNAR1**, **IL1B**, IL1RN, ISG15, MARCKS, NAMPT, NFKBIZ, OAS3, OASL, PNPT1, PPP1R15A, PTX3, RSAD2, STAT1, TNF, (APOL6, RASGEF1B)
Drulovic et al., J Neuroimmunol, 2009 [27]	**Patients:** 35 RRMS, **Treatment:** IFN-â-1b sc, **Sample:** PBMC RNA, **Platform:** real-time RT-PCR	**Good:** relapse rate reductionof >30% compared with pre-treatment and EDSS increase of less than one point after1-year therapy, **Poor:** EDSS increase and relapses	**Suggested:** 1, **Affymetrix custom:** 1, **Affymetrix original:** 1	TBX21
Eikelenboom et al., J Neuroimmunol, 2005 [28]	**Patients:** 21 RRMS, **Treatment:** none, **Sample:** T-cell surface proteins, **Platform:** FACS	no grouping, patients were evaluated by T1 lesion load changes in MRI scans performed before study onset and after several years	**Suggested:** 1, **Affymetrix custom:** 1, **Affymetrix original:** 3	ITGA4
Gilli et al., Arch Neurol, 2011 [29]	**Patients:** 101 RRMS, **Treatment:** IFN-â-1a im or sc, IFN-â-1b sc, GA, **Sample:** whole blood RNA, **Platform:** real-time RT-PCR	**Good:** administration of first-line therapy for more than 2 years of follow-up or discontinuation due to pregnancy, **Poor:** switch to second-line treatments	**Suggested:** 3, **Affymetrix custom:** 3, **Affymetrix original:** 7	NR4A2, SOCS2, TNFAIP3

Eighteen studies nominated 110 mRNA or proteins that, when measured in the blood at a single time point, may allow the prediction of an individual's course of the disease. The table provides study details, e.g., number of patients and technology used to quantify gene expression, as well as information on how long-term disease progression was evaluated. In the column “Number of Genes”, the entry “Suggested” gives for each study the number of genes that were proposed as predictive markers. “Affymetrix custom” gives the number of custom probe sets that detect the suggested genes. Custom probe sets uniquely relate to GeneCard genes and are defined by a custom CDF, which we used to preprocess our Affymetrix HG-U133 A and B microarray data. Genes for which no specific custom probe set exists are given in brackets in the rightmost column. “Affymetrix original” gives the number of probe sets according to the original CDF, which was used by Gurevich et al. to preprocess their Affymetrix HG-U133 A and A 2.0 data. Some genes are assayed by more than one probe set in the original annotation. Genes described as being predictive concordantly by more than one study are written in bold in column “List of Genes”. In addition to reassessing the prognostic value of the listed genes, we included the cytokines IL17A-E and the receptors IL17RA-E in the analysis. In total, we examined the expression signals of 112 different genes that are represented by 112 custom probe sets in our data and 204 original probe sets in the data by Gurevich et al., which we used for further validation of the results.

**Table 2:** Table 1 continued.

Publication	Study Characteristics	Clinical Groups	Number of Genes	List of Genes
Gurevich et al., BMC Med Genomics, 2009 [30]	**Patients:** 62 MS and 32 CIS, **Treatment:** IFN-â-1a im or sc, IFN-â-1b sc, GA, none, **Sample:** PBMC RNA, **Platform:** Affymetrix HG-U133 A and A 2.0	time to first relapse evaluated for a maximal period of 3.5 years. Groups: relapse within a) <500, b) >500 and <1264, c) >1264 days	**Suggested:** 23, **Affymetrix custom:** 22, **Affymetrix original:** 46	C14orf169, CA2, CLCN4, DYNC2H1, FPR2, G3BP1, IL24, KHDRBS2, LOC51145, PCOLCE2, PDCD2, PPFIA1, RHBG, SMARCA1, SPN, TAF4B, TGFB2, TP63, TRIM22, TTC28, TUBB2B, YEATS2, (ENSG00000245923)
Hagman et al., J Neuroimmunol, 2011 [31]	**Patients:** 66 MS, **Treatment:** IFN-â-1a im or sc, IFN-â-1b sc, GA, none, **Sample:** serum proteins, **Platform:** LINCOplex kit	**Good:** unchanged EDSS score after 1-year follow-up, **Poor:** EDSS increase of more than 0.5 points	**Suggested:** 2, **Affymetrix custom:** 2, **Affymetrix original:** 5	FAS, MIF
Hesse et al., Neurology, 2010 [32]	**Patients:** 23 RRMS, **Treatment:** IFN-â-1a im or sc, **Sample:** PBMC RNA, **Platform:** Affymetrix HG-Focus	**Good:** no relapse and no EDSS increase of at least one point and no MRI disease activity during 6-month follow-up, **Poor:** relapse or worsening in MRI	**Suggested:** 3, **Affymetrix custom:** 3, **Affymetrix original:** 6	IL10, TGFB1, **TNFSF10**
Lee et al., Sci Transl Med, 2011 [33]	**Patients:** 26 RRMS, **Treatment:** IFN-â-1a im or sc, IFN-â-1b sc, **Sample:** serum proteins, **Platform:** Multiplex Bead Analysis	**Good and Poor:** not exactly declared; based on the number of relapses and steroid interventions within 2-year follow-up	**Suggested:** 1, **Affymetrix custom:** 1, **Affymetrix original:** 1	IL7
Lopatinskaya et al., Mult Scler, 2006 [34]	**Patients:** 15 RRMS, **Treatment:** cM-T412, IFN-â-1a im or sc, IFN-â-1b sc, GA, **Sample:** PBMC RNA, **Platform:** real-time RT-PCR	no grouping, patients were evaluated by the increase of EDSS over a follow-up period of 10 years	**Suggested:** 2, **Affymetrix custom:** 2, **Affymetrix original:** 5	FAS, IL12A
Soilu-Hänninen et al., J Neuroimmunol, 2005 [35]	**Patients:** 24 RRMS, **Treatment:** IFN-â-1a sc, **Sample:** T-cell surface proteins, **Platform:** FACS	**Good:** unchanged or modestly increased EDSS during 4-year follow-up, **Poor:** EDSS increase of more than 1.0 points	**Suggested:** 2, **Affymetrix custom:** 2, **Affymetrix original:** 6	ITGA4, ITGB1
van Boxel-Dezaire et al., Ann Neurol, 2000 [36]	**Patients:** 26 RRMS, **Treatment:** IFN-â-1b sc, **Sample:** white blood cell RNA, **Platform:** RT-PCR	**Good and Poor:** not exactly declared; based on relapses, EDSS progression and steroid interventions within 2-year follow-up	**Suggested:** 3, **Affymetrix custom:** 3, **Affymetrix original:** 4	IL12A, IL18, TGFB1
van der Voort et al., Neurology, 2010 [37]	**Patients:** 116 RRMS, **Treatment:** IFN-â-1a im or sc, IFN-â-1b sc, GA, none, **Sample:** whole blood RNA, **Platform:** real-time RT-PCR	no grouping, patients were evaluated by time to a first new relapse	**Suggested:** 1, **Affymetrix custom:** 1, **Affymetrix original:** 1	MX1
Wandinger et al., Lancet, 2003 [38]	**Patients:** 11 RRMS, **Treatment:** IFN-â-1a sc, **Sample:** serum proteins, **Platform:** ELISA	**Good:** no relapse and no EDSS or MRI worsening after 1-year follow-up, **Poor:** one or more relapses and MRI disease activity	**Suggested:** 1, **Affymetrix custom:** 1, **Affymetrix original:** 3	**TNFSF10**

However, with a few exceptions such as NAbs to IFN, most of the suggested potentially prognostic biomarkers or marker sets have not been confirmed in a completely independent analysis. The findings of the 18 studies, which searched for blood expression markers to be measured at a single time point, are also quite inconsistent. Overall, 110 different genes have been nominated in these studies (Tables 1 and 2). Nine of those genes (IFNAR1, IFNB1, IL1B, TNFSF10, TGF-beta, ITGA4, IL10, IL12A and FAS) each were identified in two studies. Higher mRNA and protein expression levels of TNFSF10 have been found in patients who are progression-free for at least 6 months on IFN-beta treatment [32], [38]. Increased levels of IFNAR1 and IL1B were observed prior to IFN-beta therapy in non-responders by Comabella et al. [26] and recently confirmed in a subsequent publication of the same group [25]. The lack of response to this treatment has also been related to an elevated endogenous expression of IFN-beta (IFNB1) on the RNA and protein level [22], [25]. TGF-beta, ITGA4, IL10, IL12A and FAS, however, have been reported with contradicting results. For instance, TGF-beta expression levels were described as being significantly higher in patients with stable disease activity by Hesse et al. [32] but as being lower in IFN-beta responders by van Boxel-Dezaire et al. [36]. All other genes were only mentioned once. Differences in the blood fraction analyzed, measurement technology, treatment strategy, patient classification, data analysis and interpretation are factors that may account for deviations in the results.

Recently, our group published two longitudinal gene expression microarray data sets providing transcript levels in peripheral blood mononuclear cells (PBMCs) for 25 RRMS patients receiving subcutaneous (sc.) IFN-beta-1b [57], [58] and 24 RRMS patients receiving intramuscular (im.) IFN-beta-1a [59]. We followed these 49 patients for 5 years after therapy onset. In this work, we used these data and publicly available data for additional 99 patients to reassess the predictive value of the genes whose expression had been related to disease progression in the literature (Tables 1 and 2). We complemented the list with the different members of IL17 and IL17-receptor genes because they are in the spotlight of current autoimmune research [60]–[62] and increased levels of IL17F were noted in non-responders to IFN-beta therapy [22]. Possible clinical implications and functional roles of the affirmed gene markers will be discussed.

## Methods

### Study concept

So far, the expression status of 110 genes has been proposed in the literature to be an estimate of the advancement of MS (Tables 1 and 2). Different therapies were prescribed to the patients in the 18 associated studies, and the patients were typically grouped according to disease progression or therapeutic response by clinical evidence observed within two years after blood sampling. Most of the genes were also measured in the gene expression data we published lately [57]–[59]. Therefore, we used the number of relapses and changes in EDSS during a two-year follow-up to classify our 49 patients into cohorts with good and poor disease progression. We then evaluated the genes for differential expression between the groups. The list of examined genes was complemented by five IL17 and five IL17R genes. The predictive value of each gene was assessed using basic univariate statistics, even if in some of the 18 original studies very complex predictors had been built combining two or more genes even in non-linear relationships. For the subset of genes that are differentially expressed between the good and the poor outcome groups in our data, we examined whether there was further support for their clinical relevance in the expression data of other studies.

### Clinical classification of the patients

We grouped the 49 RRMS patients using different criteria. First, we allocated patients to the “very poor” group if they showed a rapid worsening of disability as reflected by an increase of more than one point in the EDSS within two years after measuring the PBMC gene expression. In addition, we defined a “poor” group, which extended the “very poor” group with patients who had at least one relapse during the two-year follow-up. The “good” group of patients was free of relapses and neurologically relatively stable. For classification of the patients, it was not important whether they were still treated with IFN-beta after two years. If so, disease progression depended on both individual course of the disease and individual benefit of the therapy. MRI scans of the brain (1.5 Tesla) were mostly performed before IFN therapy onset as well as after a few years, and were analyzed for the accumulation of T2 lesion burden independently by two experienced neurologists following the recommendations by Sailer et al. [63]. Some patients were tested for NAbs using the MX1 induction assay, but this assay was not routine. Clinical data accompany the gene expression data in the Gene Expression Omnibus (GEO) database (accession GSE19285 and GSE24427).

### Gene expression data processing

Experimental details of the time-course gene expression analysis of PBMCs from 49 patients can be found in the original articles [57]–[59]. The preprocessing of the data is described in Hecker et al. [59]. Briefly, blood samples were obtained for all patients immediately prior to the first IFN-beta injection and at different time points during IFN-beta therapy. PBMC RNA was extracted, processed, labeled and hybridized to Affymetrix HG-U133 A and B oligonucleotide arrays. A custom chip definition file (CDF) was used to preprocess the raw probe intensities with the MAS5.0 algorithm. The custom CDF was based on the information contained in the GeneAnnot database, version 1.9, and the GeneCards database, version 2.41 (http://www.xlab.unimo.it/GA_CDF/) [64]. Compared to the original CDF from Affymetrix, the custom CDF defines a set of probe sets, where each probe set contains all specific probes for one particular gene. This arrangement ensures a one-to-one correspondence between genes and custom probe sets. Data normalization was performed separately for chip types A and B and each of the two data sets by a loess fit to the data with span = 0.05 using the R package “affy”. Each A- and B-chip yielded mRNA abundances of 12175 and 7771 human genes, respectively. In this reassessment study, we only analyzed the pre-treatment (baseline) data of the predictive gene candidates (Tables 1 and 2). The full raw and processed microarray data have been deposited according to the MIAMI guidelines in the GEO database (accession GSE19285 and GSE24427). The study was approved by the University of Rostock's ethics committee and carried out according to the Declaration of Helsinki. Written informed consent was obtained from all patients before the blood sampling.

### Statistical analysis

For each gene, we compared the expression levels of the “poor” and the “very poor” group with the expression levels of the “good” group. The two-sample two-tailed t-test assuming unequal variances (t-test) was used for this purpose. As an alternative, the nonparametric two-tailed Wilcoxon rank-sum test (U test) was applied in addition if explicitly mentioned in the text. To check whether the patient groups were similar in demographic and clinical parameters, we used the t-test to examine differences in age, disease duration (months since diagnosis of definite MS) and EDSS at baseline, as well as the number of relapses in the year prior to the blood sampling. The female to male ratio was compared between the groups by Fisher's exact test. Differences in expression and patient characteristics with p-values below 0.05 were considered statistically significant. Correlation of gene levels and clinical parameters was assessed by Pearson's product-moment coefficient.

The prognostic significance of two genes, GPR3 and IL17RC, was evaluated in more detail. First, we distinguished low and high mRNA levels of these genes. For this purpose, receiver operating characteristics (ROCs) were used to compare “very poor” and “good” patients and to calculate appropriate cut-off values for defining low and high expression for both genes. Next, we investigated whether the genes' expression predicted the time until a new relapse. Using the R package “survival”, Kaplan-Meier survival curves were constructed to visualize the proportion of patients with no relapse after the blood sampling over time. Patients were grouped in this analysis according to the low and high expression of GPR3 and IL17RC, and right censoring occurred if patients withdrew from the study during the 5-year follow-up period. We tested whether the difference between the survival curves was significant by calculating the logrank test. Hazard ratios and 95% confidence intervals (CIs) were retrieved from a Cox proportional hazards model. Further, the number of patients with an increase in T2 lesion load in the “low” and “high” expression cohorts was compared using Fisher's exact test. For the subset of patients who were followed for more than 60 months in our clinic independent of the treatment, we visualized their long-term clinical course expressed in the number of relapses and the change in EDSS. We computed t-test p-values to evaluate differences in these measures between patients with low or high baseline expression of GPR3 or IL17RC. Additionally, to disclose the main and interaction effects of the patient groups and time, a linear model was fitted.

### Further validation

For those genes that were differentially expressed in our data between the “good” and “poor” or “good” and “very poor” groups, we searched for additional support of their predictive capacity. This was undertaken using publicly available data from two high-density oligonucleotide microarray studies [30], [65]. The larger data set is provided by Gurevich and coworkers [30]. They measured the PBMC gene expression in 32 clinically isolated syndrome (CIS) and 62 MS patients with Affymetrix HG-U133 A and A 2.0 arrays, and reported the time until the next relapse for each patient. Compared to our transcriptome data, they processed the raw data using the RMA algorithm and the original CDF from Affymetrix (a GeneAnnot-based custom CDF is not available for A 2.0 arrays). We downloaded the preprocessed data from GEO. The data contain information about 22215 probe sets representing roughly 13000 genes. Because the RMA method includes a log2 transformation, we calculated the antilog before conducting statistical tests on the data. CIS and MS samples were analyzed together if not otherwise declared. To compare the gene expression between patients with and without a relapse during two-year follow-up (“good” and “poor” group), we used the t-test and the U test. A gene was considered confirmed as predictive if the t-test p-value for the corresponding probe set was lower than 0.05 and if the mean difference of “good” and “poor” had the same sign in our data. Correlation, ROC and survival analyses were performed as described above.

For further verification, we took into account the microarray data published by Singh et al. [65]. The preprocessed pre-treatment data of this study were downloaded from GEO. Since a different gene expression platform was used (CodeLink UniSet Human I Bioarray), not all genes measured with Affymetrix can be found in this data set. Additionally, only five RRMS patients were studied: one long-term IFN-beta non-responder and four responders. Therefore, a gene that was expressed significantly higher/lower in the groups “poor” or “very poor” in our data was considered confirmed if the single non-responder patient showed a higher/lower expression than the other four patients. MRI measures and NAb status of IFN-treated patients were not specified in the studies by Singh et al. and Gurevich et al.

### Blood expression levels in other diseases

For the biomarker candidates that were differentially expressed in our data, we compared the PBMC transcript levels of our 49 MS patients with those of patients with a different neurological disease (chronic fatigue syndrome, CFS), patients with a different inflammatory disease (rheumatoid arthritis, RA) and healthy subjects (HS). For this purpose, we used another microarray data set generated in our group that comprises 19 pre-treatment RA samples [66], and an external public data set that comprises 8 CFS and 7 HS samples [67]. Each data set consists of Affymetrix HG-U133 A chips and was preprocessed as described above for the MS data set.

## Results

### Patient characteristics

A total of 49 MS patients were included. Two years after the initiation of IFN-beta therapy, 5 patients had stopped the treatment. One patient withdrew during this time period, resulting in the complete clinical documentation for 48 patients over two years. After 5 years, 32 patients were still under IFN-beta therapy, and 5 patients had left the study. Clinical presentation was highly variable; after the first two years, our patients had between 0 and 4 relapses (median = 0) and an EDSS-defined change in disability of between −1.0 and +4.5 (median = 0). During the two-year follow-up, 30 patients were relapse-free and had only a moderate increase in the EDSS (group “good”), and 18 patients had at least one relapse and/or a relatively strong worsening of MS symptoms (group “poor”). In the latter group, 8 patients were treated with IFN-beta-1a im., and 10 patients were treated with IFN-beta-1b sc. Four patients showed a “very poor” disease progression (1 with IFN-beta-1a im., 3 with IFN-beta-1b sc.). They had an EDSS increase of more than one point and an average of two relapses within the first two years. One of the “very poor” patients was tested for NAbs. Because of NAb positivity, he was further treated with steroids and mitoxantron. The baseline clinical characteristics were similar between the “poor” and “good” groups as well as the “very poor” and “good” groups (Table 3).

**Table 3:** Clinical and demographic characteristics of the patients.

	Patient Groups			p-values	
	Good (n = 30)	Poor (n = 18)	Very Poor (n = 4)	Poor vs Good	Very Poor vs Good
**Age (years)**	39 (±9)	36 (±11)	43 (±15)	0.435	0.668
**Gender (female:male)**	21∶9	12∶6	3∶1	1.000	1.000
**Disease duration (months)**	15 (±33)	4 (±8)	4 (±3)	0.113	0.082
**Relapses 1 year prior**	1.10 (±0.66)	0.83 (±0.62)	0.50 (±0.58)	0.167	0.125
**EDSS**	1.52 (±1.16)	1.86 (±0.95)	2.25 (±1.19)	0.271	0.313

Patients were grouped based on relapses and EDSS changes during a two-year follow-up period. Pre-treatment parameters were similar between these groups, although disease duration tended to be longer for the “good” patient group. Mean ± standard deviation and t-test p-values are given for age, disease duration, EDSS at baseline and the number of relapses in the year before the gene expression measurement. The female to male ratio was compared by Fisher's exact test.

### Genes predictive of disease progression

Of the 110 predictive gene candidates from the 18 studies (Tables 1 and 2) and the 10 additional IL17-related genes, 112 genes were measured by the Affymetrix microarray set using the GeneAnnot-based custom CDF. Hence, 8 genes could not be analyzed with our data. The expression of 104 genes was determined with the A-chip. For the remaining 8 genes we used the signal intensities from the B-chip.

Significantly differential expression was found for five genes between the “poor” and “good” groups, and 12 genes were differentially expressed between the “very poor” and “good” groups (Table 4). GPR3 and IL1RN were identified in both comparisons. A high statistical significance (p-value<0.001) was only achieved by three genes (GPR3, IL17RC and TUBB2B), and no gene was capable of a 100% accurate separation of the clinical groups. Interestingly, IL17RA and IL17RC appeared as new prognostic marker candidates in this analysis.

**Table 4:** Differentially expressed genes and further validation.

Gene		Goertsches et al., 2010 [57]; Hecker et al., 2010 [59]				Gurevich et al., 2009 [30]		Singh et al., 2007 [65]	
Symbol	Reference	GeneCard	Poor vs Good	Very Poor vs Good	Difference	Probe Set	Validation	Probe	Validation
CA11	Achiron et al., 2007 [21]	GC19M053833	0.020	ns	Poor > Good	209726_at		NM_001217	ok
CA2	Gurevich et al., 2009 [30]	GC08P086563	ns	0.007	Good > Very Poor	209301_at	ok	NM_000067	ok
CLCN4	Gurevich et al., 2009 [30]	GC0XP010085	ns	0.046	Good > Very Poor	205149_s_at	ok	AF052117	
DNM1	Achiron et al., 2007 [21]	GC09P130005	ns	0.022	Good > Very Poor	215116_s_at	ok	NM_004408	ok
FPR2	Gurevich et al., 2009 [30]	GC19P056955	ns	0.022	Good > Very Poor	210772_at		————	————
GPR3	Achiron et al., 2007 [21]	GC01P027591	0.018	<0.001	Good > Poor	214613_at	ok	NM_005281	ok
IL1RN	Comabella et al., 2009 [26]	GC02P113591	0.042	0.012	Good > Poor	212659_s_at		NM_000577	
IL7	Lee et al., 2011 [33]	GC08M079807	ns	0.038	Very Poor > Good	206693_at		NM_000880	
NAMPT	Comabella et al., 2009 [26]	GC07M105675	ns	0.006	Good > Very Poor	217739_s_at		————	————
PPFIA1	Gurevich et al., 2009 [30]	GC11P069794	0.040	ns	Poor > Good	202066_at	ok	————	————
RRN3	Achiron et al., 2007 [21]	GC16M015061	ns	0.012	Very Poor > Good	222204_s_at	ok	NM_018427	
TUBB2B	Gurevich et al., 2009 [30]	GC06M003172	ns	<0.001	Good > Very Poor	209372_x_at		————	————
YEATS2	Gurevich et al., 2009 [30]	GC03P184899	0.009	ns	Poor > Good	221203_s_at	ok	AB033023	ok
IL17RA	————	GC22P015947	ns	0.006	Good > Very Poor	205707_at		NM_014339	
IL17RC	————	GC03P009933	ns	<0.001	Good > Very Poor	64440_at	ok	————	————

In total, 15 of the 112 examined genes were significantly differentially expressed between the “poor” and “good” or “very poor” and “good” patient cohorts in our microarray data. Thirteen of those genes were proposed as potentially prognostic blood biomarkers in earlier studies, as stated in the “Reference” column. Two genes, IL17RC and IL17RA, have not been directly linked to long-term disease progression so far but are believed to play important roles in the context of autoimmunity. They were expressed at lower levels in the “very poor” group. The custom probe set (GeneCard) and t-test p-values are given for the analysis of our data. We validated the results using the data from Gurevich et al. and Singh et al. A gene was considered confirmed by their data if it was differentially expressed between patients with moderate and severe disease courses in the same manner as in our data. The respective Affymetrix original probe sets and CodeLink Bioarray probes used for the quantification of the genes' expression are given in the table. Five of the 15 genes were not measured in the study by Singh et al. ns  =  not significant. ok  =  confirmed.

The PBMC expression levels of the shortlisted genes were then compared between MS, other diseases (CFS and RA) and healthy individuals. We found evidence for higher levels of IL1RN, FPR2 and IL17RC, and lower levels of IL7 in MS and RA samples compared to CFS and HS samples (Supporting Information S1). The different data sets used for this analysis showed a similar distribution of signal intensities, however, they were generated in different labs and even small differences in the experimental protocols may have lead to biases. Therefore, we did not perform additional statistical tests to compare the transcript levels across the different disorders.

Next, we included the data from Gurevich et al. to further validate the biomarker candidates. A relapse occurred in 51 of their patients (“poor” group, 14 CIS and 37 MS patients), while 43 patients remained relapse-free over two years (“good” group). The two groups were matched in age and gender (t-test and Fisher's exact test p-value>0.05, respectively), and 17 of the patients started an IFN-beta therapy during the follow-up period [30]. Of the 15 genes that were differentially expressed in our data, we were able to confirm 8 genes with the data from Gurevich et al. (Table 4). Five of these genes were expressed at significantly lower levels, and 3 genes were expressed at significantly higher levels in the PBMCs of patients with poor disease progression.

Ten of the 15 genes were also measured in the study by Singh et al. [65]. For 5 genes, the only non-responder in their data set had the lowest/highest expression when the gene was expressed at a lower/higher level in the “very poor” or “poor” cohort of our patients. Transcript levels of 9 genes were verified as being predictive of prognosis either by the data of Gurevich et al. or by the data of Singh et al. (Table 4). Four of these genes (GPR3, CA11, DNM1 and RRN3) were originally proposed to distinguish good and poor outcomes by Achiron et al. [21], and another four of these genes (CA2, PPFIA1, CLCN4 and YEATS2) were suggested to be markers in the publication by Gurevich et al. [30]. For the latter four genes, we expected that we would be able to “confirm” their differential expression when we re-used the data from Gurevich et al. for further validation.

**Figure 1:**
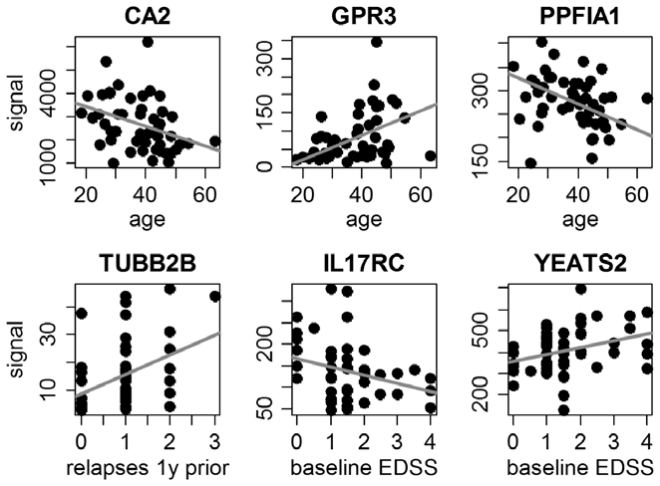
Correlation of gene expression and clinical parameters. The transcript levels of six genes correlated significantly with clinical or demographic data of the patients (Pearson's correlation p-value<0.05). For instance, GPR3 expression correlated positively with age, and IL17RC expression correlated negatively with EDSS. Orthogonal linear regression lines are shown in gray. y  =  year.

Signal intensities of six of the genes correlated significantly with clinical parameters (Figure 1); for example, GPR3 expression correlated with the age of our patients (R = 0.341, p-value = 0.016), and IL17RC expression correlated negatively with EDSS at study onset (R = −0.304, p-value = 0.034).

### Two promising markers: GPR3 and IL17RC

**Figure 2:**
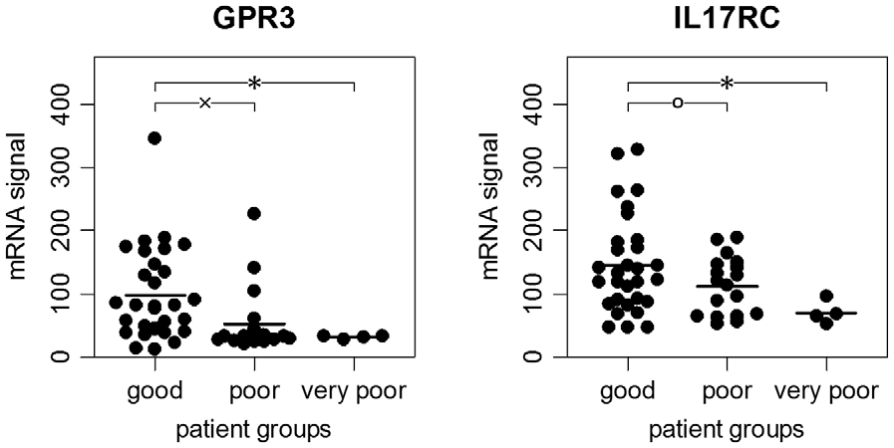
GPR3 and IL17RC mRNA expression in PBMCs of MS patients. In our microarray data, the mRNA levels of GPR3 and IL17RC were significantly higher in patients with “good” (n = 30) disease outcomes than in patients with “poor” (n = 18) or “very poor” (n = 4) outcomes after a two-year follow-up. Patients in the “very poor” group are a subset of the “poor” group. When comparing patients with “poor” and “good” disease progression, there is a marked overlap in the IL17RC signal intensities, but there is still a tendency toward differential expression (p = 0.068). The horizontal black lines represent the means. The figure was drawn using the function “ehplot” of the R package “plotrix”. ^ο^ p<0.10, ^x^ p<0.05, * p<0.005 by t-test.

Two genes emerged as the most predictive potential biomarkers. GPR3 and IL17RC were repeatedly and significantly differentially expressed, including when the U test was used. Their enrichment was as follows: a) they were expressed at significantly lower levels in our data in patients with worse clinical outcomes; b) they were expressed at significantly lower levels in the “poor” group of the Gurevich et al. study; and c) in the latter, they were also expressed at significantly lower levels in patients with at least one relapse during the two-year follow-up when analyzing the 32 CIS and 62 MS subjects independently (Supporting Information S1). Furthermore, in the data from Singh et al., the GPR3 mRNA signal was lower in the non-responder than in the four responders. GPR3 and IL17RC both encode cell surface receptors and are relatively weakly expressed in PBMCs (Figure 2, Supporting Information S1).

Cut-off values to define low and high signal intensities for the genes were determined using ROC analysis. For our data, a GPR3 signal of <34 and an IL17RC signal of <70 was considered low. While 15 of our 49 patients had “low” GPR3 levels, 10 of these patients had “low” IL17RC levels prior to treatment with IFN-beta. Analogously, for the data from Gurevich et al. (that were preprocessed differently, as explained in the methods), a GPR3 signal of <50.5 and an IL17RC signal of <117.5 were considered low (Supporting Information S1). There were 73 out of 94 patients with “low” GPR3 levels and 53 patients with “low” IL17RC levels.

**Figure 3:**
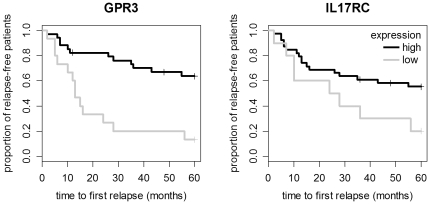
Kaplan-Meier survival curves for low and high expression of GPR3 and IL17RC. Survival curves were used to visualize the proportion of relapse-free patients from the baseline up through a 5-year follow-up. Of the 49 patients included, 34 had “high” and 15 had “low” GPR3 levels. Similarly, 39 patients had “high” and 10 had “low” IL17RC levels. Small vertical tick marks indicate losses, where a patient's survival time has been right-censored (n = 3). More than half of the patients in the group with low GPR3 expression had a relapse within 13 months after the blood sampling and the start of IFN-beta therapy. Low expression of the two receptors appears to correlate with a higher risk of relapses.

Kaplan-Meier survival analyses with censoring were then performed on both data sets. In our data, 3 patients were censored because they were relapse-free after therapy onset and left the study during the 5-year follow-up period. Patients with low GPR3 levels had a significantly shorter time until the next relapse in our data (logrank test p-value = 0.0001; hazard ratio 4.3, CI: 1.9–9.6) as well as in the data from Gurevich et al. (p-value = 0.0006; hazard ratio 3.4, CI: 1.6–7.2). We also compared the survival curves of patients with low and high IL17RC signals. The difference was significant in the patient cohort from Gurevich et al. (p-value = 0.0009; hazard ratio 2.35, CI: 1.4–4.0) but slightly above the significance threshold for our patients (p-value = 0.0521; hazard ratio 2.3, CI: 1.0–5.2) (Figure 3, Supporting Information S1). We conclude that higher PBMC transcript levels of both genes are associated with a lower risk of MS relapses.

**Figure 4:**
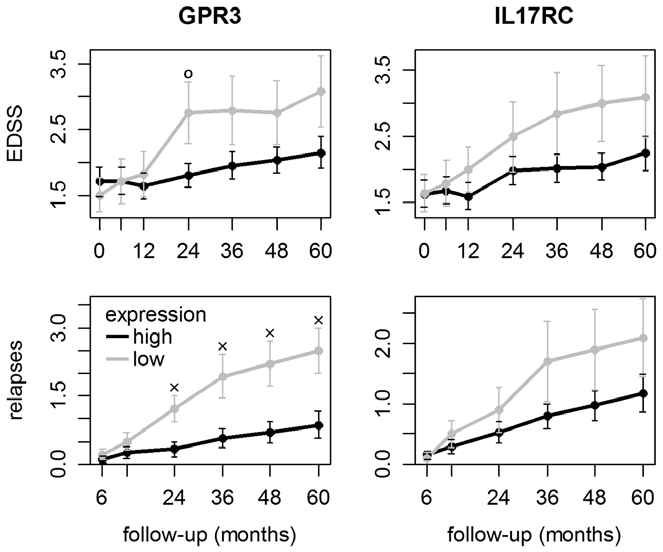
EDSS progression and relapses for patients with low and high expression of GPR3 and IL17RC. We have the complete clinical data from 5 years for 44 MS patients. Patients with low baseline expression of GPR3 (n = 14) or IL17RC (n = 10) tended to have a higher rate of relapses and a stronger increase in the EDSS. Higher levels of these receptors in PBMCs may therefore be favorable. Error bars indicate the standard error. ^ο^ p<0.10, ^x^ p<0.05 by t-test.

We have complete clinical information for at least 5 years for 44 of our 49 patients. For those patients, the mean EDSS and the mean cumulative number of relapses from baseline to year 5 are visualized in figure 4, with patients grouped according to low and high expression of GPR3 and IL17RC. Linear regression revealed a significant interaction effect between the patient groups and time for GPR3 (EDSS p-value = 0.0194, relapses p-value = 0.0003) and a tendency for such an interaction effect for IL17RC (EDSS p-value = 0.0702, relapses p-value = 0.0523). For 41 patients, MRI scans were performed before as well as 3-6 years after the start of therapy. The formation of new T2 lesions was observed in 11 of 13 patients with low GPR3 expression and 15 of 28 patients with high GPR3 expression (p-value = 0.0836).

GPR3 and IL17RC expression is correlated in our data (R = 0.409, p-value = 0.0036) as well as in the data from the Gurevich et al. study (R = 0.359, p-value = 0.0004). Therefore, using both genes may not necessarily yield an improvement in the prediction of clinical progression, but a detailed multivariate analysis is beyond the scope of this study.

## Discussion

In the past few years, several studies have proposed gene markers whose blood expression status could allow neurologists to predict the long-term progression of disability and relapses in patients with MS (Tables 1 and 2). Only nine genes were reported twice, and five of those had conflicting results (see introduction). Reasons for such divergent findings might be the relatively small patient cohorts examined in some of these studies, the variability that results from the patients' heterogeneity and the different treatments employed (including co-medications), and the differences in molecular biological and bioinformatic tools used. Another issue could be that the currently identified biomarker candidates have only a moderate discriminative power. To reassess the proposed markers, we compared in PBMC the amounts of mRNA, which not necessarily strongly correlate with the amounts of active protein, between patients with good and poor disease progression during two years of follow-up. Differential expression was tested with the t-test and a significance level of alpha = 0.05. This p-value cut-off is quite liberal because 112 genes were evaluated. However, this less conservative statistic lowers the chance that we miss potential markers and, therefore, confers higher statistical power. As a limitation, our approach could not verify combinatorial or non-linear associations. We also did not exclude genes with very low signal intensities from the analysis, even if it appeared that some genes could not be detected with the Affymetrix microarrays, e.g., TUBB2B (Figure 1, Supporting Information S1).

Only 15 genes were differentially expressed between the “poor”, “very poor” and “good” groups of our 49 patients (Table 4). A few genes showed very low p-values and were also confirmed by further validation. For the latter, we used the data from Gurevich et al. [30] and Singh et al. [65]. To our knowledge, these are the only publicly available oligonucleotide microarray data sets in the field [19], [20] other than ours and studies that do not (or do not yet) provide clinical follow-up information [68]–[70]. Gurevich et al. used their data to estimate the time until the next relapse by constructing complex predictors based on the expression of up to 10 genes. CA2 is one of the genes included in those predictors. It was verified to have lower expression levels in patients with strong disease progression in our data and the data from Singh et al. Interestingly, CA2 has been found lower expressed than in controls years before the onset of MS [71].

We noticed a higher expression of IL1RN in MS patients with “good” clinical outcomes in our data. The average expression of IL1RN was elevated compared to samples of healthy individuals, but not compared to samples of RA patients (Supporting Information S1). IL1RN inhibits the activity of the pro-inflammatory cytokines IL1A and IL1B by binding to their receptor. The ratio of these molecules is therefore assumed to be critical for systemic and brain inflammation [72]. Treatment with GA has been shown to enhance IL1RN blood levels in MS patients, mainly by affecting monocytes. Therefore, GA triggers less of an inflammatory response [73]. IL1RN is also up-regulated in the blood in response to IFN-beta treatment [74]. Further, IL1RN gene therapy improves the course of experimental autoimmune encephalomyelitis, the animal model of MS [75].

Some patients showed an increased expression of typical IFN-induced genes prior to the treatment with IFN-beta (data not shown). This may indicate an elevated endogenous type I IFN-activity in individual patients. There is currently much discussion on whether this phenomenon is associated with better or worse therapeutic outcomes [25], [26], [37], [47]. TNFSF10 is an IFN-responsive gene and was nominated as a predictive marker by Wandinger et al. [38] and Hesse et al. [32]. High levels of the endogenously produced IFN-beta itself and its receptor IFNAR1 have been linked to therapy non-response as well [22], [25], [26]. However, in our data, we could not find a differential expression of TNFSF10, IFNB1 or IFNAR1 in patients with more severe disease progression, and we also did not find differences in the data from Gurevich et al. (data not shown). Moreover, none of the other reassessed prominent IFN-induced biomarkers, such as MX1, IFIT1, IFI44 and OASL, reached statistical significance when we compared the expression levels of patients with good or poor long-term clinical outcomes. Therefore, we could not confirm that the signaling of IFN can predict the course of MS and responses to IFN-beta administration. This may be due to the experimental design of our study, e.g., we evaluated the RNA levels in PBMC, while the monocyte cell surface protein level of IFNAR1 was described to be predictive [25], [26]. Sensitivity limits of the Affymetrix microarrays may also explain why we could not validate some of the potential blood biomarkers, e.g., IFNB1 transcripts were virtually not detectable (average signal intensity <15).

One of the most promising prognostic genes that emerged from this analysis was IL17RC. IL17RC and IL17RA were both expressed more weakly in the “very poor” group of our patients, and further validation using the data by Gurevich et al. confirmed the differential expression of IL17RC. Both membrane receptors have not been directly associated with MS disease activity or progression so far. They were shown to be overexpressed in whole blood of RA patients [76], which is consistent with our results (Supporting Information S1), and in MS, IL17RA expression in PBMCs was described as being modulated during IFN-beta therapy in responders [39]. IL17RC is expressed at much lower levels than IL17RA in blood cells such as monocytes [76]. Functionally, they form a complex to mediate the effects of IL17A and IL17F homodimers, as well as IL17A/F heterodimers [60], [77]. In MS patients treated with IFN-beta, higher IL17F protein concentrations were detected in the serum of a subset of therapy non-responders [22]. However, we did not observe a differential expression of IL17F at the mRNA level in our analyses. IL17A and IL17F are mainly produced by a subset of CD4+ T cells (Th17 cells) and have been linked to a variety of inflammatory and autoimmune conditions [62]. IL17RA and IL17RC are essential for the signaling of these cytokines and have likely no intrinsic activity [78]. It is tempting to speculate why patients with lower IL17RC expression showed a more severe MS progression during the follow-up period. The cellular composition of the blood may be different in those patients in a way that they have lower numbers of IL17RC producing cells. On the other hand, the expression of this receptor in these cells may be less effective. Interestingly, some of the many splice forms of IL17RC suggest the existence of soluble receptors, which may act as decoy receptors [79]. Higher levels of IL17RC may therefore be beneficial if the cells release the receptor to dampen IL17A/F signaling. The ratio of IL17F and soluble and membrane-bound IL17RC may be important in MS. This opens novel therapeutic avenues; a soluble version of IL17RC is currently being developed as a potential drug for MS [80], while another clinical trial (NCT01051817) is investigating the effects of a monoclonal antibody that targets IL17A (AIN457).

The other promising gene marker is GPR3, which also encodes a receptor. GPR3 was evaluated because it was described as being down-regulated in RRMS patients who have a poor disease course [21], and has therefore been patented for the prediction of MS prognosis by Achiron and Gurevich [81]. We verified this differential expression in our data, the data by Gurevich et al. and the data by Singh et al. The attained p-values were <0.01 according to both the t-test and the U test. Hence, four independent data sets attest to GPR3 a predictive value, although PBMC transcript levels are quite low. GPR3 is a G protein-coupled membrane receptor predominantly expressed in the human brain and testis. It constitutively activates G protein G(alpha)s. As a result, adenylate cyclase is activated and therefore cAMP production is stimulated [82]. The overexpression of GPR3 in neurons increases amyloid-beta production, which is pathologically deposited in Alzheimer's disease [83]. The sphingosine-1-phosphate (S1P) receptors are close phylogenetic neighbors of GPR3 [84]. S1P ligands enhance Ca2+ release from GPR3 expressing cells; therefore, GPR3 is assumed to be subject to modulation by S1P [85]. S1Ps are hormone-like lipid signaling molecules that are involved in many biological processes, including platelet activation and protection of endothelial cells from apoptosis [86]. Intriguingly, a novel class of MS drugs resembles naturally occurring S1P; Fingolimod (FTY720), the first oral agent for MS, acts as a S1P receptor modulator. The compound is phosphorylated to form fingolimod-phosphate, which binds to S1P receptors and leads to the down-regulation of lymphocyte egress from lymph nodes into the circulation. This is assumed to reduce systemic inflammation and lymphocyte infiltration into the CNS. In addition, fingolimod may also have direct CNS effects because it can cross the blood-brain barrier [87]. Similar drugs are currently in phase II clinical trials (NCT00879658, NCT01006265). However, to our knowledge, it is unknown whether GPR3 is affected by these treatments – either directly or indirectly.

More studies are needed to further clarify the ligand-dependent functional roles of the different IL17 receptors and the different S1P or closely related lipid receptors in the blood and brain. We may better understand disease and treatment mechanisms if we have more data on the cell type-specific expression of IL17RC and GPR3. Specific experiments on DNA polymorphisms and mRNA splice variants of these genes and on their respective protein levels should help disclose their biological significance. This, together with a more detailed analysis of their regulation by transcription factors and regulatory RNAs (e.g., microRNAs as important post-transcriptional regulators [88]) and their interactions with ligands and other molecules, may even enhance their potential as prognostic biomarkers for MS.

In this study, we could not ascertain to what extent the genes are markers of a clinical response to IFN-beta therapy. While all of our 49 patients and all 5 patients in the Singh et al. study started treatment with IFN-beta immediately after the blood sampling, only 17 of the 94 patients in the Gurevich et al. study initiated this therapy during follow-up. Therefore, low levels of GPR3 and IL17RC may indicate a higher inherent disease activity, which may be perceived as a suboptimal therapy response. This also implies that we cannot exclude a beneficial therapeutic effect in patients with poor outcome, at least for IFN NAb-negative patients. The relationship of the genes' expression and therapeutic benefit thus remains an open question. Nevertheless, GPR3 and/or IL17RC will be of help in individual treatment decisions, if they can be further confirmed and established as markers of future disease burden. This requires larger studies with long clinical follow-up periods. Meanwhile, the need for reliable prognostic markers is growing as more treatments with different efficacies and risks enter the MS drug market.

In conclusion, of the 110 genes that have been proposed in the literature as predictive markers of future clinical or MRI disease activity, most could not be confirmed in our reassessment analysis, which was based on PBMC RNA levels. However, GPR3 was significantly lower expressed in patients with long-term poor disease progression in all data sets. GPR3 is therefore the best supported biomarker. We also investigated the IL17 cytokines and receptors and propose IL17RC as a novel, promising transcript-based biomarker candidate. GPR3 and IL17RC may have the potential to facilitate improved patient care, but larger studies employing more sensitive RNA detection methods are required to further examine the predictive value of their blood expression levels. In addition, we may better understand the pathophysiology of MS if we better understand the specific roles of these receptors.

## Supporting Information

Supporting Information S1 This document includes details of the additional validation using the data from Gurevich et al. as well as a comparison of PBMC gene expression levels in MS, two other diseases and healthy controls.
(PDF

## References

[1] Compston A, Coles A (2008). Multiple sclerosis.. Lancet.

[2] Sawcer S, Hellenthal G, Pirinen M, Spencer CC, Patsopoulos NA (2011). Genetic risk and a primary role for cell-mediated immune mechanisms in multiple sclerosis.. Nature.

[3] Sospedra M, Martin R (2005). Immunology of multiple sclerosis.. Annu Rev Immunol.

[4] Vosoughi R, Freedman MS (2010). Therapy of MS.. Clin Neurol Neurosurg.

[5] Mendes A, Sá MJ (2011). Classical immunomodulatory therapy in multiple sclerosis: how it acts, how it works.. Arq Neuropsiquiatr.

[6] Rudick RA, Polman CH (2009). Current approaches to the identification and management of breakthrough disease in patients with multiple sclerosis.. Lancet Neurol.

[7] Debouverie M, Pittion-Vouyovitch S, Louis S, Guillemin F; LORSEP Group (2008). Natural history of multiple sclerosis in a population-based cohort.. Eur J Neurol.

[8] Langer-Gould A, Popat RA, Huang SM, Cobb K, Fontoura P (2006). Clinical and demographic predictors of long-term disability in patients with relapsing-remitting multiple sclerosis: a systematic review.. Arch Neurol.

[9] Mowry EM, Pesic M, Grimes B, Deen S, Bacchetti P (2009). Demyelinating events in early multiple sclerosis have inherent severity and recovery.. Neurology.

[10] Tintoré M, Rovira A, Río J, Nos C, Grivé E (2006). Baseline MRI predicts future attacks and disability in clinically isolated syndromes.. Neurology.

[11] Neema M, Stankiewicz J, Arora A, Guss ZD, Bakshi R (2007). MRI in multiple sclerosis: what's inside the toolbox?. Neurotherapeutics.

[12] Fisniku LK, Brex PA, Altmann DR, Miszkiel KA, Benton CE (2008). Disability and T2 MRI lesions: a 20-year follow-up of patients with relapse onset of multiple sclerosis.. Brain.

[13] Graber JJ, Dhib-Jalbut S (2011). Biomarkers of disease activity in multiple sclerosis.. J Neurol Sci.

[14] Giovannoni G, Munschauer FE, Deisenhammer F (2002). Neutralising antibodies to interferon beta during the treatment of multiple sclerosis.. J Neurol Neurosurg Psychiatry.

[15] Deisenhammer F, Schellekens H, Bertolotto A (2004). Measurement of neutralizing antibodies to interferon beta in patients with multiple sclerosis.. J Neurol.

[16] Malucchi S, Gilli F, Caldano M, Marnetto F, Valentino P (2008). Predictive markers for response to interferon therapy in patients with multiple sclerosis.. Neurology.

[17] Killestein J, Polman CH (2011). Determinants of interferon-beta efficacy in patients with multiple sclerosis.. Nat Rev Neurol.

[18] Sbardella E, Tomassini V, Gasperini C, Bellomi F, Cefaro LA (2009). Neutralizing antibodies explain the poor clinical response to interferon beta in a small proportion of patients with multiple sclerosis: a retrospective study.. BMC Neurol.

[19] Goertsches RH, Zettl UK, Hecker M (2011). Sieving treatment biomarkers from blood gene-expression profiles: a pharmacogenomic update on two types of multiple sclerosis therapy.. Pharmacogenomics.

[20] Kemppinen AK, Kaprio J, Palotie A, Saarela J (2011). Systematic review of genome-wide expression studies in multiple sclerosis.. BMJ Open.

[21] Achiron A, Gurevich M, Snir Y, Segal E, Mandel M (2007). Zinc-ion binding and cytokine activity regulation pathways predicts outcome in relapsing-remitting multiple sclerosis.. Clin Exp Immunol.

[22] Axtell RC, de Jong BA, Boniface K, van der Voort LF, Bhat R (2010). T helper type 1 and 17 cells determine efficacy of interferon-beta in multiple sclerosis and experimental encephalomyelitis.. Nat Med.

[23] Baranzini SE, Mousavi P, Rio J, Caillier SJ, Stillman A (2005). Transcription-based prediction of response to IFNbeta using supervised computational methods.. PLoS Biol.

[24] Bartosik-Psujek H, Stelmasiak Z (2006). The interleukin-10 levels as a potential indicator of positive response to interferon beta treatment of multiple sclerosis patients.. Clin Neurol Neurosurg.

[25] Bustamante MF, Fissolo N, Río J, Espejo C, Costa C (2011). Implication of the toll-like receptor 4 pathway in the response to interferon-beta in multiple sclerosis.. Ann Neurol.

[26] Comabella M, Lünemann JD, Río J, Sánchez A, López C (2009). A type I interferon signature in monocytes is associated with poor response to interferon-beta in multiple sclerosis.. Brain.

[27] Drulovic J, Savic E, Pekmezovic T, Mesaros S, Stojsavljevic N (2009). Expression of Th1 and Th17 cytokines and transcription factors in multiple sclerosis patients: does baseline T-bet mRNA predict the response to interferon-beta treatment?. J Neuroimmunol.

[28] Eikelenboom MJ, Killestein J, Izeboud T, Kalkers NF, Baars PA (2005). Expression of adhesion molecules on peripheral lymphocytes predicts future lesion development in MS.. J Neuroimmunol.

[29] Gilli F, Navone ND, Perga S, Marnetto F, Caldano M (2011). Loss of braking signals during inflammation: a factor affecting the development and disease course of multiple sclerosis.. Arch Neurol.

[30] Gurevich M, Tuller T, Rubinstein U, Or-Bach R, Achiron A (2009). Prediction of acute multiple sclerosis relapses by transcription levels of peripheral blood cells.. BMC Med Genomics.

[31] Hagman S, Raunio M, Rossi M, Dastidar P, Elovaara I (2011). Disease-associated inflammatory biomarker profiles in blood in different subtypes of multiple sclerosis: Prospective clinical and MRI follow-up study.. J Neuroimmunol.

[32] Hesse D, Krakauer M, Lund H, Søndergaard HB, Langkilde A (2010). Breakthrough disease during interferon-[beta] therapy in MS: No signs of impaired biologic response.. Neurology.

[33] Lee LF, Axtell R, Tu GH, Logronio K, Dilley J (2011). IL-7 promotes T(H)1 development and serum IL-7 predicts clinical response to interferon-beta in multiple sclerosis.. Sci Transl Med.

[34] Lopatinskaya L, Zwemmer J, Uitdehaag B, Lucas K, Polman C (2006). Mediators of apoptosis Fas and FasL predict disability progression in multiple sclerosis over a period of 10 years.. Mult Scler.

[35] Soilu-Hänninen M, Laaksonen M, Hänninen A, Erälinna JP, Panelius M (2005). Downregulation of VLA-4 on T cells as a marker of long term treatment response to interferon beta-1a in MS.. J Neuroimmunol.

[36] van Boxel-Dezaire AH, van Trigt-Hoff SC, Killestein J, Schrijver HM, van Houwelingen JC (2000). Contrasting responses to interferon beta-1b treatment in relapsing-remitting multiple sclerosis: does baseline interleukin-12p35 messenger RNA predict the efficacy of treatment?. Ann Neurol.

[37] van der Voort LF, Vennegoor A, Visser A, Knol DL, Uitdehaag BM (2010). Spontaneous MxA mRNA level predicts relapses in patients with recently diagnosed MS.. Neurology.

[38] Wandinger KP, Lünemann JD, Wengert O, Bellmann-Strobl J, Aktas O (2003). TNF-related apoptosis inducing ligand (TRAIL) as a potential response marker for interferon-beta treatment in multiple sclerosis.. Lancet.

[39] Stürzebecher S, Wandinger KP, Rosenwald A, Sathyamoorthy M, Tzou A (2003). Expression profiling identifies responder and non-responder phenotypes to interferon-beta in multiple sclerosis.. Brain.

[40] Satoh J, Nakanishi M, Koike F, Onoue H, Aranami T (2006). T cell gene expression profiling identifies distinct subgroups of Japanese multiple sclerosis patients.. J Neuroimmunol.

[41] Graber JJ, Ford D, Zhan M, Francis G, Panitch H (2007). Cytokine changes during interferon-beta therapy in multiple sclerosis: correlations with interferon dose and MRI response.. J Neuroimmunol.

[42] Weinstock-Guttman B, Bhasi K, Badgett D, Tamaño-Blanco M, Minhas M (2008). Genomic effects of once-weekly, intramuscular interferon-beta1a treatment after the first dose and on chronic dosing: Relationships to 5-year clinical outcomes in multiple sclerosis patients.. J Neuroimmunol.

[43] Rudick RA, Rani MR, Xu Y, Lee JC, Na J (2011). Excessive biologic response to IFN-beta is associated with poor treatment response in patients with multiple sclerosis.. PLoS One.

[44] Caggiula M, Batocchi AP, Frisullo G, Angelucci F, Patanella AK (2005). Neurotrophic factors and clinical recovery in relapsing-remitting multiple sclerosis.. Scand J Immunol.

[45] Vogt MH, Floris S, Killestein J, Knol DL, Smits M (2004). Osteopontin levels and increased disease activity in relapsing-remitting multiple sclerosis patients.. J Neuroimmunol.

[46] Waubant E, Goodkin DE, Gee L, Bacchetti P, Sloan R (1999). Serum MMP-9 and TIMP-1 levels are related to MRI activity in relapsing multiple sclerosis.. Neurology.

[47] Hesse D, Krakauer M, Lund H, Søndergaard HB, Limborg SJ (2011). Disease protection and interleukin-10 induction by endogenous interferon-beta in multiple sclerosis?. Eur J Neurol.

[48] van Baarsen LG, Vosslamber S, Tijssen M, Baggen JM, van der Voort LF (2008). Pharmacogenomics of interferon-beta therapy in multiple sclerosis: baseline IFN signature determines pharmacological differences between patients.. PLoS One.

[49] Khademi M, Kockum I, Andersson ML, Iacobaeus E, Brundin L (2011). Cerebrospinal fluid CXCL13 in multiple sclerosis: a suggestive prognostic marker for the disease course.. Mult Scler.

[50] Sharief MK, Hentges R (1991). Association between tumor necrosis factor-alpha and disease progression in patients with multiple sclerosis.. N Engl J Med.

[51] Simpson S, Taylor B, Blizzard L, Ponsonby AL, Pittas F (2010). Higher 25-hydroxyvitamin D is associated with lower relapse risk in multiple sclerosis.. Ann Neurol.

[52] Vandenbroeck K, Urcelay E, Comabella M (2010). IFN-beta pharmacogenomics in multiple sclerosis.. Pharmacogenomics.

[53] DeLuca GC, Ramagopalan SV, Herrera BM, Dyment DA, Lincoln MR (2007). An extremes of outcome strategy provides evidence that multiple sclerosis severity is determined by alleles at the HLA-DRB1 locus.. Proc Natl Acad Sci U S A.

[54] Freedman MS, Laks J, Dotan N, Altstock RT, Dukler A (2009). Anti-alpha-glucose-based glycan IgM antibodies predict relapse activity in multiple sclerosis after the first neurological event.. Mult Scler.

[55] Villar LM, Masjuan J, González-Porqué P, Plaza J, Sádaba MC (2003). Intrathecal IgM synthesis is a prognostic factor in multiple sclerosis.. Ann Neurol.

[56] Villar LM, Sádaba MC, Roldán E, Masjuan J, González-Porqué P (2005). Intrathecal synthesis of oligoclonal IgM against myelin lipids predicts an aggressive disease course in MS.. J Clin Invest.

[57] Goertsches RH, Hecker M, Koczan D, Serrano-Fernandez P, Moeller S (2010). Long-term genome-wide blood RNA expression profiles yield novel molecular response candidates for IFN-beta-1b treatment in relapsing remitting MS.. Pharmacogenomics.

[58] Serrano-Fernández P, Möller S, Goertsches R, Fiedler H, Koczan D (2010). Time course transcriptomics of IFNB1b drug therapy in multiple sclerosis.. Autoimmunity.

[59] Hecker M, Goertsches RH, Fatum C, Koczan D, Thiesen HJ (2012). Network analysis of transcriptional regulation in response to intramuscular interferon-beta-1a multiple sclerosis treatment.. Pharmacogenomics J.

[60] Gaffen SL (2009). Structure and signalling in the IL-17 receptor family.. Nat Rev Immunol.

[61] Hu Y, Shen F, Crellin NK, Ouyang W (2011). The IL-17 pathway as a major therapeutic target in autoimmune diseases.. Ann N Y Acad Sci.

[62] Quesniaux V, Ryffel B, Di Padova F (2009). Th 17 cells: role in inflammation and autoimmune disease..

[63] Sailer M, Fazekas F, Gass A, Kappos L, Radue EW (2008). Cerebral and spinal MRI examination in patients with clinically isolated syndrome and definite multiple sclerosis.. Rofo.

[64] Ferrari F, Bortoluzzi S, Coppe A, Sirota A, Safran M (2007). Novel definition files for human GeneChips based on GeneAnnot.. BMC Bioinformatics.

[65] Singh MK, Scott TF, LaFramboise WA, Hu FZ, Post JC (2007). Gene expression changes in peripheral blood mononuclear cells from multiple sclerosis patients undergoing beta-interferon therapy.. J Neurol Sci.

[66] Koczan D, Drynda S, Hecker M, Drynda A, Guthke R (2008). Molecular discrimination of responders and nonresponders to anti-TNF alpha therapy in rheumatoid arthritis by etanercept.. Arthritis Res Ther.

[67] Gow JW, Hagan S, Herzyk P, Cannon C, Behan PO (2009). A gene signature for post-infectious chronic fatigue syndrome.. BMC Med Genomics.

[68] De Jager PL, Jia X, Wang J, de Bakker PI, Ottoboni L (2009). Meta-analysis of genome scans and replication identify CD6, IRF8 and TNFRSF1A as new multiple sclerosis susceptibility loci.. Nat Genet.

[69] Fernald GH, Knott S, Pachner A, Caillier SJ, Narayan K (2007). Genome-wide network analysis reveals the global properties of IFN-beta immediate transcriptional effects in humans.. J Immunol.

[70] Gandhi KS, McKay FC, Cox M, Riveros C, Armstrong N (2010). The multiple sclerosis whole blood mRNA transcriptome and genetic associations indicate dysregulation of specific T cell pathways in pathogenesis.. Hum Mol Genet.

[71] Achiron A, Grotto I, Balicer R, Magalashvili D, Feldman A (2010). Microarray analysis identifies altered regulation of nuclear receptor family members in the pre-disease state of multiple sclerosis.. Neurobiol Dis.

[72] Spulber S, Bartfai T, Schultzberg M (2009). IL-1/IL-1ra balance in the brain revisited - evidence from transgenic mouse models.. Brain Behav Immun.

[73] Burger D, Molnarfi N, Weber MS, Brandt KJ, Benkhoucha M (2009). Glatiramer acetate increases IL-1 receptor antagonist but decreases T cell-induced IL-1beta in human monocytes and multiple sclerosis.. Proc Natl Acad Sci U S A.

[74] Goertsches RH, Hecker M, Zettl UK (2008). Monitoring of multiple sclerosis immunotherapy: from single candidates to biomarker networks.. J Neurol.

[75] Furlan R, Bergami A, Brambilla E, Butti E, De Simoni MG (2007). HSV-1-mediated IL-1 receptor antagonist gene therapy ameliorates MOG(35-55)-induced experimental autoimmune encephalomyelitis in C57BL/6 mice.. Gene Ther.

[76] Zrioual S, Toh ML, Tournadre A, Zhou Y, Cazalis MA (2008). IL-17RA and IL-17RC receptors are essential for IL-17A-induced ELR+ CXC chemokine expression in synoviocytes and are overexpressed in rheumatoid blood.. J Immunol.

[77] Kuestner RE, Taft DW, Haran A, Brandt CS, Brender T (2007). Identification of the IL-17 receptor related molecule IL-17RC as the receptor for IL-17F.. J Immunol.

[78] Ho AW, Gaffen SL (2010). IL-17RC: a partner in IL-17 signaling and beyond.. Semin Immunopathol.

[79] Haudenschild D, Moseley T, Rose L, Reddi AH (2002). Soluble and transmembrane isoforms of novel interleukin-17 receptor-like protein by RNA splicing and expression in prostate cancer.. J Biol Chem.

[80] Power C, Magnenat L, inventors; Laboratoires Serono SA, assignee (2008). Soluble IL-17RC variant and uses thereof.. United States patent.

[81] Achiron A, Gurevich M, inventors; Hashomer medical research infrastructure, assignee (2010). Methods and kits for predicting prognosis of multiple sclerosis.. United States patent.

[82] Tanaka S, Ishii K, Kasai K, Yoon SO, Saeki Y (2007). Neural expression of G protein-coupled receptors GPR3, GPR6, and GPR12 up-regulates cyclic AMP levels and promotes neurite outgrowth.. J Biol Chem.

[83] Thathiah A, Spittaels K, Hoffmann M, Staes M, Cohen A (2009). The orphan G protein-coupled receptor 3 modulates amyloid-beta peptide generation in neurons.. Science.

[84] Kostenis E (2004). Novel clusters of receptors for sphingosine-1-phosphate, sphingosylphosphorylcholine, and (lyso)-phosphatidic acid: new receptors for “old” ligands.. J Cell Biochem.

[85] Uhlenbrock K, Gassenhuber H, Kostenis E (2002). Sphingosine 1-phosphate is a ligand of the human gpr3, gpr6 and gpr12 family of constitutively active G protein-coupled receptors.. Cell Signal.

[86] Young N, Van Brocklyn JR (2006). Signal transduction of sphingosine-1-phosphate G protein-coupled receptors.. ScientificWorldJournal.

[87] Chun J, Hartung HP (2010). Mechanism of action of oral fingolimod (FTY720) in multiple sclerosis.. Clin Neuropharmacol.

[88] Thamilarasan M, Koczan D, Hecker M, Paap B, Zettl UK (2011). MicroRNAs in multiple sclerosis and experimental autoimmune encephalomyelitis.. Autoimmun Rev.

