# Supplementary material for: Reassessment of Blood Gene Expression Markers for the Prognosis of Relapsing-Remitting Multiple Sclerosis
Source: PLoS One. 2011 Dec 27;6(12):e29648. doi: 10.1371/journal.pone.0029648 (PMC3246503; doi:10.1371/journal.pone.0029648)
Supplement: Supporting Information S1 — This document includes details of the additional validation using the data from Gurevich et al. as well as a comparison of PBMC gene expression levels in MS, two other diseases and healthy controls. (PDF [file pone.0029648.s001.pdf]

Reassessment of Blood Gene Expression Markers for the Prognosis of Relapsing-Remitting Multiple Sclerosis

Supplementary Material

Michael Hecker[1], Brigitte Katrin Paap[2], Robert Hermann Goertsches[1], Ole Kandulski[2], Christian Fatum[2], Dirk Koczan[3], Hans-Peter Hartung[4], Hans-Juergen Thiesen[3], Uwe Klaus Zettl[2]

[1] Steinbeis Transfer Center for Proteome Analysis, Schillingallee 68, 18057 Rostock, Germany, e-mail: michael.hecker@rocketmail.com, phone: +49 381 494-5891, fax: +49 381 494-5882

[2] University of Rostock, Department of Neurology, Gehlsheimer Str. 20, 18147 Rostock, Germany

[3] University of Rostock, Institute of Immunology, Schillingallee 70, 18055 Rostock, Germany

[4] Heinrich Heine University, Department of Neurology, Moorenstr. 5, 40225 Düsseldorf, Germany

Content

We reassessed potentially prognostic blood biomarkers in multiple sclerosis. This supplementary material provides results from the further validation using the Affymetrix microarray data from Gurevich et al. (BMC Med Genomics, 2009). The data provide PBMC transcript levels of 32 CIS and 62 MS patients. We grouped patients with "poor" and "good" disease course depending on whether they had at least one documented relapse within two years after the blood sampling. Additionally, for the 15 genes that were differentially expressed in our data between patient groups with different clinical response status, we compared the PBMC expression levels to those of patients with chronic fatigue syndrome, rheumatoid arthritis and healthy individuals.

Table S1: Mean gene expression levels in different diseases and healthy subjects.

		Multiple sclerosis	Rheumatoid arthritis	Chronic fatigue syndrome	Healthy subjects
**Data set**	**Accession**	GSE19285 & 24427	E-MTAB-11	GSE14577	GSE14577
	**Individuals**	49	19	8	7
**Data distribution**	**Range**	[0,42247]	[0,34162]	[0,31530]	[0,31695]
	**Mean**	665	655	669	668
	**Median**	229	213	202	200
**GeneCard**	**Symbol**				
GC19M053833	CA11	262 (±90)	224 (±85)	222 (±74)	193 (±43)
GC08P086563	CA2	2673 (±1137)	1510 (±781)	3154 (±1059)	2601 (±1989)
GC0XP010085	CLCN4	66 (±31)	64 (±33)	104 (±40)	97 (±38)
GC09P130005	DNM1	194 (±65)	163 (±74)	186 (±48)	152 (±51)
GC19P056955	FPR2	855 (±219)	887 (±384)	179 (±96)	160 (±38)
GC01P027591	GPR3	81 (±69)	72 (±49)	44 (±30)	60 (±39)
GC02P113591	IL1RN	525 (±143)	658 (±573)	232 (±23)	247 (±43)
GC08M079807	IL7	57 (±20)	53 (±25)	104 (±28)	85 (±12)
GC07M105675	NAMPT	1425 (±451)	1330 (±953)	977 (±472)	611 (±317)
GC11P069794	PPFIA1	277 (±53)	261 (±42)	295 (±43)	290 (±49)
GC16M015061	RRN3	257 (±77)	252 (±82)	433 (±73)	487 (±192)
GC06M003172	TUBB2B	16 (±12)	23 (±15)	28 (±19)	22 (±17)
GC03P184899	YEATS2	408 (±107)	372 (±59)	598 (±54)	596 (±57)
GC22P015947	IL17RA	2448 (±575)	2351 (±532)	1429 (±405)	1798 (±643)
GC03P009933	IL17RC	135 (±69)	97 (±47)	35 (±11)	42 (±5)

In our data, 15 of the 112 evaluated biomarker candidate genes were significantly higher or lower expressed in patients with worse disease progression than in patients having no relapse and no strongly increased EDSS during follow-up (see main text). For those genes, we compared the PBMC transcript levels of our 49 MS patients with those of patients with chronic fatigue syndrome (CFS), patients with rheumatoid arthritis (RA) and healthy individuals (HS). For this purpose, we used another microarray data set generated in our group (Koczan et al., Arthritis Res Ther, 2008) and an external data set (Gow et al., BMC Med Genomics, 2009). Each data set consists of Affymetrix HG-U133 A chips that were preprocessed uniformly to provide the expression levels of 12175 genes (see main text). The accessions of the GEO and ArrayExpress databases are given in the table as well as minimum, maximum, mean and median of the measured signal intensities per data set, and mean ± standard deviation for each gene. Red, yellow and white cell colors represent high, medium and low mRNA amounts, respectively. The table indicates higher levels of IL17RC, IL1RN and FPR2, and lower levels of IL7 in MS and RA samples compared to CFS and HS samples. However, since the data for CFS and HS were obtained by another lab, systematic differences between the data sets are likely and thus the results should be interpreted with caution.

Table S2: Differential expression in patients with good and poor outcome.

Gene Symbol	ProbeSet	All (n=94) Poor: 51, Good: 43	CIS (n=32) Poor: 14, Good: 18	MS (n=62) Poor: 37, Good: 25	Difference
CA11	209726_at	0.074	0.447	0.006	Good > Poor
CA2	209301_at	0.003	0.301	0.009	Good > Poor
CLCN4	205149_s_at	<0.001	<0.001	<0.001	Good > Poor
DNM1	215116_s_at	0.039	0.193	0.118	Good > Poor
FPR2	210772_at	0.616	0.536	0.361	———
GPR3	214613_at	<0.001	0.030	0.007	Good > Poor
IL1RN	212659_s_at	0.208	0.220	0.450	———
IL7	206693_at	0.375	0.837	0.208	———
NAMPT	217739_s_at	0.249	0.116	0.943	———
PPFIA1	202066_at	0.007	0.054	0.034	Poor > Good
RRN3	222204_s_at	0.067	0.041	0.400	Poor > Good
TUBB2B	209372_x_at	0.084	0.488	0.099	———
YEATS2	221203_s_at	<0.001	0.059	<0.001	Poor > Good
IL17RA	205707_at	0.430	0.464	0.198	———
IL17RC	64440_at	<0.001	0.005	0.020	Good > Poor

The 15 shortlisted genes were reanalyzed using the data by Gurevich et al. In these data, 43 of the patients (18 CIS and 25 MS patients) remained relapse-free for about two years (group "good"). Their PBMC mRNA levels were compared to those patients having at least one relapse in this period of time (group "poor"). Shown are the U test p-values for these comparisons, and p-values<0.05 are highlighted in orange. In contrast to the other genes, IL17RA and IL17RC have not been directly related to disease progression or therapy outcome before our study, and are therefore given at the bottom of this table. The column "Difference" specifies, which of the patient groups had higher expression signals.

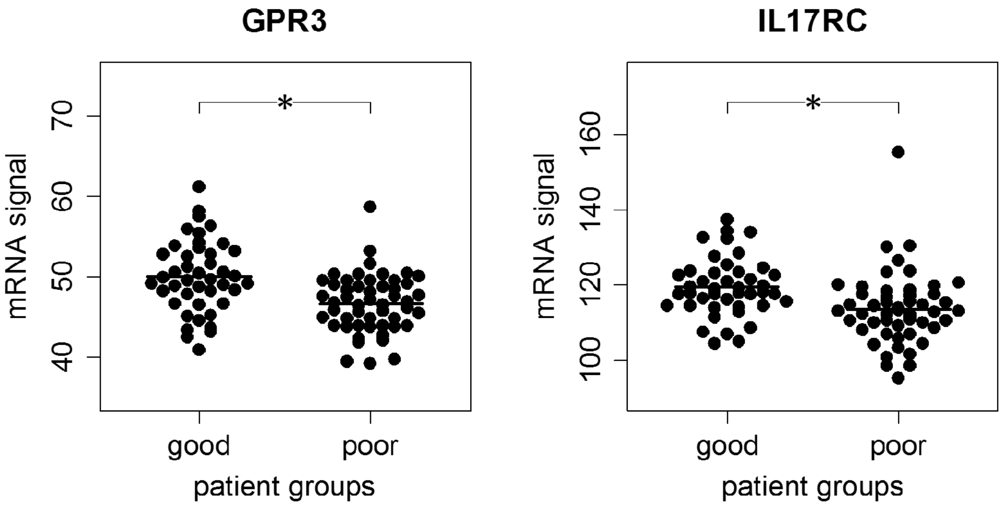

Figure S1: GPR3 and IL17RC mRNA expression in PBMC of 94 patients as determined by microarray analysis by Gurevich et al. Levels of both genes were significantly lower in those patients who had at least one relapse within two years of follow-up (n=51, group "poor"). Though, there is a considerable overlap in the signal intensities of both clinical groups. Means are presented by horizontal black lines. The figure was drawn using the function "ehplot" of R package "plotrix". * $p<0.005$ by t-test.

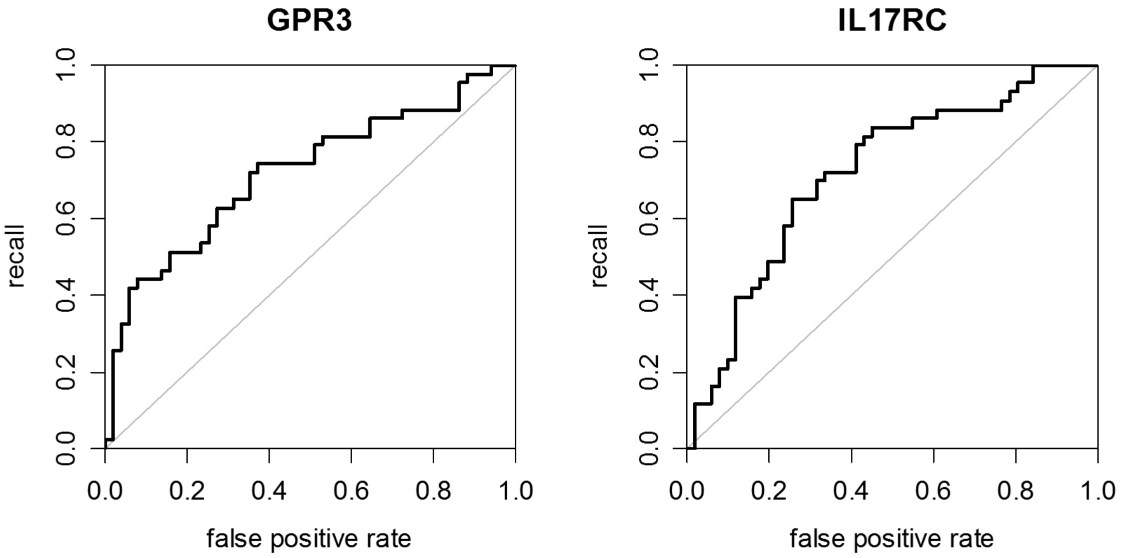

Figure S2: ROC curve analyses of GPR3 and IL17RC for prognosis of disease progression based on the data by Gurevich et al. The ROC curves visualize the accuracies of GPR3 and IL17RC as biomarkers for distinguishing patients with "good" (n=43) and "poor" (n=51) course of disease. For an arbitrary signal cut-off, the ROC curve displays the false positive rate (1 - specificity) against the recall (sensitivity). The better the predictions, the closer the resulting curve will be to the upper-left corner. We used the ROC curves to determine appropriate signal cut-off values defining "low" and "high" expression for both genes. The area under the curve is 0.719 in case of GPR3, and 0.721 in case of IL17RC.

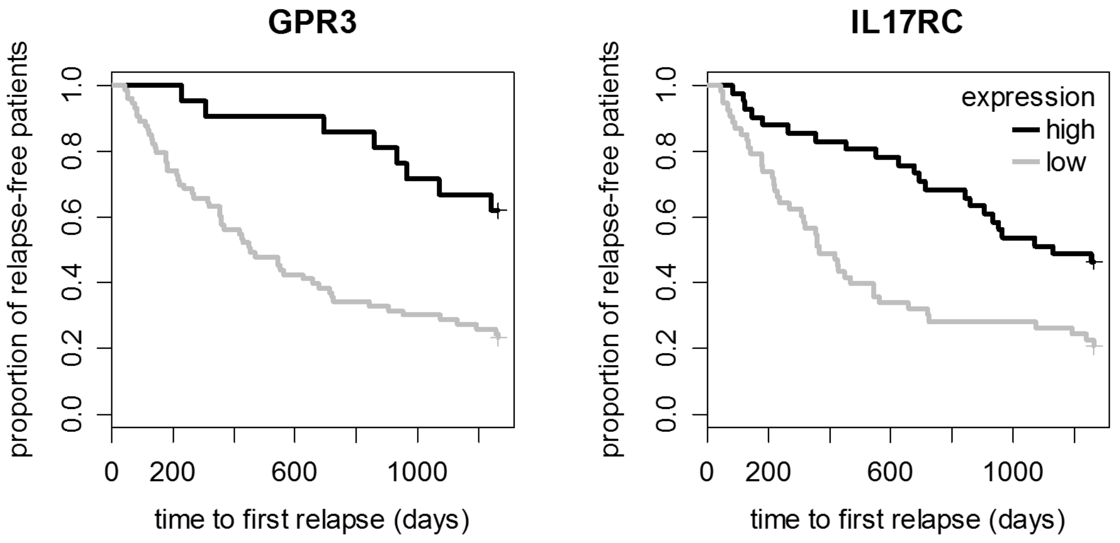

Figure S3: Kaplan-Meier survival curves for low and high expression of GPR3 and IL17RC. The curves visualize the proportion of relapse-free patients after the blood sampling. Of the 94 patients included in the study by Gurevich et al., 21 had "high" and 73 had "low" GPR3 levels. Analogously, 41 patients had "high" and 53 had "low" IL17RC levels. The differences between the survival curves of patients with low and high expression were significant according to the logrank test for GPR3 (p-value=0.0006) as well as for IL17RC (p-value=0.0009). Hazard ratio and 95% confidence interval retrieved from a Cox proportional hazards model were 3.4 (1.6-7.2) for GPR3, and 2.35 (1.4-4.0) for IL17RC. According to this, low expression of GPR3 and IL17RC is associated with a shorter time to the next relapse.
